# Genetic Diversity of *Borreliaceae* Species Detected in Natural Populations of *Ixodes ricinus* Ticks in Northern Poland

**DOI:** 10.3390/life13040972

**Published:** 2023-04-09

**Authors:** Beata Wodecka, Valentyna Kolomiiets

**Affiliations:** Department of Genetics and Genomics, Institute of Biology, Szczecin University, 71-415 Szczecin, Poland

**Keywords:** *Borreliaceae*, *Borreliella*, *Borrelia*, genetic diversity, *Ixodes ricinus*

## Abstract

In Europe, *Ixodes ricinus* tick is the vector of Lyme disease spirochetes and their relatives (*Borreliella* genus) and *Borrelia miyamotoi*. However, a newly described tick *I. inopinatus* with similar biological features and separated from *I. ricinus* may act as a vector for different *Borrelia* species. To date, eleven *Borreliella* species were detected in the natural populations of *I. ricinus*. Recently, two North American species have been detected in ticks parasitizing bats and red foxes in Europe, i.e., *B. lanei* and *B. californiensis* pointing to the necessity for searching for them in natural tick populations. In this study, using the *coxI* molecular marker only *I. ricinus* was identified in field-collected ticks with the exception of individual specimens of *Haemaphysalis concinna*. Using the *flaB* gene and *mag*-*trnI* intergenic spacer as molecular markers 14 *Borreliaceae* species have been detected with various frequencies in different parts of northern Poland. Among infected ticks, the most frequent were *Borreliella* (*Bl.*) *afzelii* (29.4%) and *Bl. garinii* (20.0%), followed by *Bl. spielmanii*, *Bl. valaisiana*, *Bl. lanei*, *Bl. californiensis*, *B. miyamotoi*, *Bl. burgdorferi*, *Bl. carolinensis*, *Bl. americana*, *B. turcica*, *Bl. lusitaniae*, *Bl. bissettiae* and *Bl. finlandensis*. Three of the above-mentioned species, i.e., *Bl. lanei*, *Bl. californiensis* and *B. turcica* were detected in this study for the first time in the natural ixodid tick population in Europe. The existence of the newly detected spirochetes increases their total diversity in Europe and points to the necessity of careful identification and establishment of the actual distribution of all *Borreliaceae* species transmitted by *I. ricinus*.

## 1. Introduction

One of the most common tick-borne diseases within the Northern Hemisphere is Lyme borreliosis (LB) caused by spirochetes belonging to *Borreliella* genus [1,2], formerly *Borrelia burgdorferi* sensu lato (s.l.) complex, the bacteria belonging to *Borreliaceae* family [3]. The European incidence of LB consists of more than 200,000 annual cases with considerable regional differences [4]. In Poland, there were over 20,000 LB cases in 2019 [5]. The principal vector of LB spirochetes in Europe is the common tick (*Ixodes ricinus*) which is found mainly in deciduous and mixed forests willingly treated by humans as recreational places [6].

To date, 14 different species of the *Borreliella* genus have been reported in Europe, but only eleven have been detected in host seeking *I. ricinus*, i.e., *Borreliella (Bl.) garinii*, *Bl. afzelii*, *Bl. burgdorferi*, *Bl. valaisiana*, *Bl. lusitaniae*, *Bl. bissettii*, *Bl. spielmanii*, *Bl. bavariensis*, *Bl. finlandensis*, *Bl. carolinensis* and *Bl. americana* [7,8,9,10]. The twelfth is *Bl. turdi,* detected only in *I. ricinus* collected from birds [9,11,12]. *Bl. finlandensis*, *Bl. carolinensis*, *Bl. turdi*, and *Bl. americana* are not considered pathogenic for humans [13]. Two other *Borreliella* species, i.e., *Bl. lanei* and *Bl. californiensis* were recently described in ticks collected from bats in Poland and Romania and from red foxes in Poland [14,15].

*Borreliella afzelii* and *Bl. garinii* are the most frequent species in ticks and the most causative factors of LB in humans [13,16]. The two mentioned species are associated in Europe with specific clinical manifestations: *Bl. garinii* with neuroborreliosis and *Bl. afzelii* with acrodermatitis chronica atrophicans, but also may include less specific symptoms [17]. *Bl. burgdorferi* seems to be the most causative species in North America with the symptoms of Lyme arthritis and Lyme neuroborreliosis; in Europe, it is the species of less concern [13]. The other five *Borreliella* species possess pathogenic potential, that is *Bl. valaisiana*, *Bl. lusitaniae*, *Bl. bissettiae*, *Bl. spielmanii*, and *Bl. bavariensis* [13].

Additionally, in Europe *I. ricinus* is also a vector tick for another *Borreliaceae* family member of the genus *Borrelia*, i.e., *B. miyamotoi*, classified as relapsing fever-like spirochetes [18] and causing Borrelia miyamotoi disease, BMD [19]. *B. miyamotoi* is reported in Europe for the last twenty years [18,20,21,22,23]. The symptoms of BMD consist mainly of relapsing fever and non-specific flu-like illness but less specific neurological symptoms are also reported [24].

Recently, North American studies on the modified serological procedure for the detection of antibodies of LB and relapsing fever (RF) causative agents demonstrated in patients with symptoms of tick-borne diseases, the presence not only of *Bl. burgdorferi* antibodies but also other pathogenic and non-pathogenic *Borreliella* and *Borrelia* species [25]. Among the pathogenic were the European LB species *Bl. afzelii*, *Bl. garinii* and *Bl. spielmanii* but also *B. miyamotoi*—the relapsing fever-like spirochete causing BMD. Among the non-pathogenic *Borreliaceae* species detected in patients with tick-borne disease symptoms were *Bl. californiensis* and *B. turcica* [25]. *Bl. californiensis* is the fourteenth LB species detected in Europe but only in ticks infesting foxes and the individual tick infected with *B. turcica* was also reported in this study [15]. According to above-mentioned data among the 14 *Borreliella* species detected in Europe *Bl. finlandensis*, *Bl. carolinensis*, *B. turdi*, *Bl. lanei*, and *Bl. americana* were not isolated from humans [13,25,26].

The knowledge about the local distribution of *Borreliaceae* species is crucial for the risk assessment of infection; the study of Fesler et al. [25] mentioned above points to the new species with pathogenic potential. Therefore, the most important aspect in the study of *Borreliaceae* species distribution in ticks populations is their precise differentiation to make the possible distinction between pathogenic and non-pathogenic species and point to the most prevalent in the studied localities. For this reason, the aim of the study was the assessment of the number and the prevalence of *Borreliaceae* species in natural populations of ticks in seven recreational localities of northern Poland. Two molecular markers described elsewhere [15] were used to make the identification of *Borreliaceae* species precise and reliable.

## 2. Material and Methods

**Figure 1:**
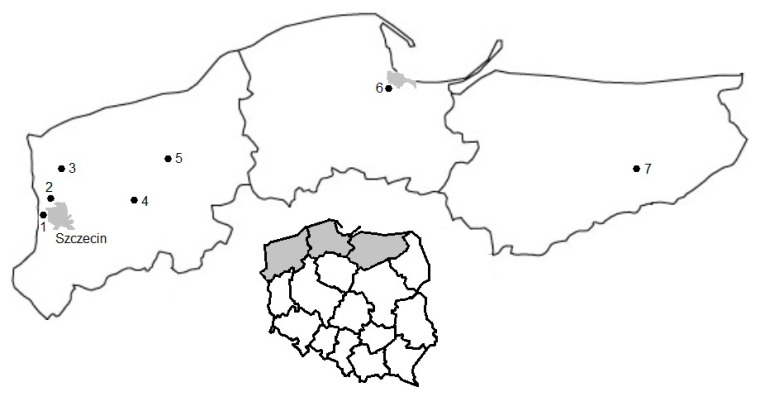
*Ixodes ricinus* tick collection sites in northern Poland. 1—Lubieszyn, 2—Bartoszewo, 3—Zielonczyn, 4—Ciemnik, 5—Świerznica, 6—Gdańsk, 7—Bełdany Lake.

**Study sites and collection of *Ixodes ricinus*.** Ticks were collected on seven localities in northern Poland including three voivodships localized in the humid continental climatic zone with warm summers: West Pomerania (five study sites), Pomerania and Warmia-Masuria (one study site each, Figure 1). Study sites in West Pomerania were located inside four forest complexes: Wkrzańska Forest (Bartoszewo and Lubieszyn), Goleniów Forest (Zielonczyn), Ińsko Landscape Park (Ciemnik) and Drawsko Landscape Park (Świerznica, Figure 1). In the Pomerania voivodship, the study site was located in Tricity Landscape Park containing the southern part of Gdańsk. In the Warmia-Masuria voivodship, the locality of the study site was on the west shore of Bełdany Lake (Figure 1). All mentioned localities consisted of mixed forest formed mainly by common oak (*Quercus robur*), European beech (*Fagus sylvatica*), Norway spruce (*Picea abies*), Scots pine (*Pinus sylvestris*) and a well-developed understory. The localities are also recreational areas for the nearby inhabitants and are often visited by strollers and mushroom pickers.

In total, 2188 specimens of ticks were collected, including 96 females, 132 males, 1524 nymphs and 436 larvae. Ticks were collected once from each study site by sweeping up the vegetation up to 1 m with a flannel flag during the middle of May in the years 2016 (Bartoszewo, Bełdany Lake, Zielonczyn) and 2017 (Ciemnik, Gdańsk, Lubieszyn, Świerznica). Ticks were removed from the flag with tweezers, placed in Eppendorf tubes containing 70% ethanol and stored at −20 °C until the next study.

**Morphological tick identification.** As morphological identification of adult ticks is easier than immature stages, females and males were determined using the taxonomic keys by Siuda [27] and Estrada-Pena et al. [28]. Molecular procedures were then used for verifying and confirming adult tick morphological identification and for the species determination of immature stages.

**DNA extraction.** DNA extraction from host-seeking ticks was performed with a phenol-chloroform protocol [29]. All tick individuals (larva, nymph or adult) were crushed through high-speed shaking (50 Hz for 5 min) in plastic tubes with stainless steel suspended in 100 mL of PBS buffer using TissueLyser LT (Qiagen, Hilden, Germany). Next, 500 mL of 29 buffer (0.19 M NH4Cl, 0.011 M KHCO3 and 0.024 M EDTA), 100 mL of Lysis buffer (0.017 M SDS, 0.01 M TRIS, 0.01 M EDTA) and 1 mL of Proteinase K (20 mg/mL) (BioShop, Burlington, ON, Canada) were added. Subsequently, ticks were placed in a 56 °C water bath for 3 h. Following the incubation, 300 mL of phenol (BioShop) was added, and the tube was vortexed for 30 s and centrifuged for 10 min at 9000 rpm. The supernatant was transferred successively to three additional tubes containing 400 mL of phenol–chloroform (1:1) and 300 mL of chloroform (POCH, Gliwice, Poland) (twice), then vortexed for 30 s and centrifuged for 10 min at 9000 rpm. Finally, the supernatant was transferred to the last tube, and DNA was precipitated by adding 500 mL of isopropanol (POCH). The pellet was rinsed with 70% ethanol and air-dried before suspension in Tris–EDTA (TE) buffer (pH 8.0). DNA samples were stored at −70 °C before PCR analyses.

**Molecular tick identification.** To confirm the accuracy of morphological tick identification according to taxonomic keys, a nested PCR assay based on the mitochondrial cytochrome c oxidase subunit I (*coxI*) molecular marker and primer sets was used, as described elsewhere [15] (Table 1).

In the first stage, including all tick specimens, molecular identification was performed based on PCR-restriction fragment length polymorphism analysis (PCR-RFLP). PCR-amplified sequences of the *coxI* gene generated with primers CO1-375F and CO1-1086R were digested with enzyme HpyF3I (Thermo Fisher Scientific, Waltham, MA, USA) to obtain RFLP patterns for different Ixodidae species. In the second stage, to validate the identification conducted by PCR-RFLP analysis of the *coxI* gene, partial sequencing of Ixodidae DNA of *coxI* fragments amplified with inner primer sets CO1-375F and CO1-1086R was performed for a subset of amplicons representing different restriction patterns. Sequencing was conducted in Macrogen Europe (Amsterdam, The Netherlands). Representative partial sequences (n = 10) were deposited in GenBank. The *coxI* sequences are listed as follows: OP882699-OP882707 (*I. ricinus*), OP882708 (*Haemaphysalis concinna*).

We conducted the detection of *Borreliaceae* spirochetes DNA by nested PCR and species identification by the PCR-RFLP procedure and sequence length polymorphism. Nested PCR procedures with two primer sets were used to detect the *flaB* gene fragments and the intergenic spacer fragments between *mag* and *trnI* genes of *Borreliaceae* spirochetes [15,20] (Table 1). In each PCR run, DNA was isolated from one of the reference strains representing *Bl. burgdorferi* IRS, *Bl. garinii* 20047, *Bl. afzelii* VS461, *Bl. valaisiana* VS116, *Bl. bissettii* DN127, *Bl. spielmanii* PC-Eq17, *Bl. californiensis* CA446, *Bl. carolinensis* SCW-22, *Bl. lanei* CA28, *Bl. americana* CA8 or *B. turcica* IST7 (German Collection of Microorganisms and Cell Cultures—DSMZ, Leibniz, Germany) was used as the positive control and TE buffer as a negative control. The PCR products were separated on 1.5% agarose gel (Bioshop, Boston, MA, USA) and archived as described elsewhere [30]. The DNA fragments of the *flaB* gene amplified with primers 220f and 823r were digested with enzyme HpyF3I (Thermo Fisher Scientific, Waltham, MA, USA) to differentiate the RFLP patterns of *Borrelia* species as described earlier [31] and one of specified, i.e., PsuI, SatI or VspI (Thermo Fisher Scientific, Table 2). As the modification of using the Ecl136II restriction enzyme [31], PsuI allowed differentiating between *Bl. garinii* and *Bl. bavariensis*, SatI enabled distinguishing *Bl. burgdorferi* not only from *Bl. finlandensis* but also from *Bl. americana* and VspI was used to differentiate *Bl. californiensis* and *Bl. turdi* (Table 2). The DNA fragments of *mag*-*trnI* intergenic spacer amplified with primers glz435f and ile65r represent different lengths of sequence depending on *Borreliaceae* species (Table 1).

***Borreliaceae* DNA sequencing, sequences alignment and analysis of genetic diversity.** Partial sequencing of *flaB* gene fragments obtained with primer pairs FL84F/FL976R and FL120F/FL908R and *mag*-*trnI* intergenic spacer fragments of *Borreliaceae* family members (Table 1) was performed for positive amplicons. DNA sequencing was performed in Macrogen Europe (Netherlands). Sequencing analyses revealed distinct patterns representing different species of the *Borreliaceae* family.

One hundred and one *flaB* gene sequences and eighty sequences of the *mag*-*trnI* intergenic spacer of *Borreliaceae* spirochetes were deposited in GenBank. The *flaB* gene sequences are listed as follows: MK604253-MK604264 (*Bl. garinii*), MK604265-MK604271 (*Bl. afzelii*), MK604272-MK604273, OP879323-OP879325 (*Bl. burgdorferi*), MK604274-MK604286, OP879326-OP879329 (*Bl. valaisiana*), MK604287-MK604288 (*Bl. lusitaniae*), MK604289-MK604300, OP879330 (*Bl. spielmanii*), MZ146997-MZ146998 (*Bl. bissettiae*), MK604301 (*Bl. finlandensis*), MK604302-MK604304, OP879331 (*Bl. californiensis*), MK604305-MK604312 (*Bl. carolinensis*), MK604313-MK604329 (*Bl. lanei*), MK604330-MK604331 (*Bl. americana*), MK604332, OP879332-OP879333 (*B. turcica*), MK604451-MK604458 (*B. miyamotoi*). The sequences of *mag*-*trnI* intergenic spacer are listed as follows: MZ146969-MZ146972, OP879334-OP879336 (*Bl. garinii*), MZ146928-MZ149941 (*Bl. afzelii*), MZ146947-MZ146951, OP879337 (*Bl. burgdorferi*), MK604274-MK604286, OP879338-OP879340 (*Bl. valaisiana*), MZ146973-MZ146974 (*Bl. lusitaniae*), MZ146978-MZ146979, OP879341-OP879344 (*Bl. spielmanii*), MZ146952-MZ146955 (*Bl. bissettiae*), MZ146965-MZ146968 (*Bl. finlandensis*), MZ146956-MZ146962 (*Bl. californiensis*), MZ146963-MZ146964 (*Bl. carolinensis*), MZ146975-MZ146977 (*Bl. lanei*), MZ146942-MZ146946 (*Bl. americana*), MZ146993-MZ146996 (*B. turcica*), MZ146983-MZ146992 (*B. miyamotoi*).

The obtained sequences were compared to those of reference strains from GenBank (Table 3). Aligned sequences representing *flaB* gene fragments and the *mag-trnI* intergenic spacer of the aforementioned *Borreliaceae* strains were examined with MEGA 11 software (Molecular Evolutionary Genetics Analysis, version 11) [32]. Relationships between individuals were assessed by distance estimation between sequences as a measure of the number of allelic substitutions on selected loci. They were obtained by dividing the number of nucleotide differences by the total number of nucleotides compared. Distance values were measured as the mean distance within each group representing separate species (a measure of diversity inside a species) and as the mean distance between species (a measure of diversity between two species). Distances were computed with the Tamura 3-parameter model using a maximum likelihood (ML) method [32] with 1000 bootstrap replicates. The MEGA analytic tool of model selection was applied to choose the best model of DNA analysis and the Tamura 3-parameter model was used to compute the distance values and construct ML phylogenetic tree on the basis of the *flaB* gene or Hasegawa–Kishino–Yano model to construct the tree on the basis of *mag-trnI* intergenic spacer.

**Statistical analyses.** *Borreliaceae* spirochetes prevalence was analyzed in ticks using the chi-square test with Yates’ correction. Differences in mean intensity of tick infestation in the Mann–Whitney U test with *p* < 0.05 were considered statistically significant. All calculations were conducted using Statistica 8.0 software (StatSoft Inc., Tulsa, OK, USA).

## 3. Results

**Identification of tick species collected in northern Poland.** Among 2188 ixodid ticks collected (96 females, 132 males, 1524 nymphs and 436 larvae) 2187 represented *I. ricinus* species and one nymph *Haemaphysalis concinna* according to the molecular data obtained on the basis of *coxI* gene PCR-RFLP analysis and sequencing.

**Detection of *Borreliaceae* DNA in field collected *I. ricinus*.** The DNA of *Borreliaceae* spirochetes was detected in 416 of 2187 *I. ricinus* (19.0%) and the DNA of spirochetes was detected at all study sites ranging from 8.3% to 32.6% (Table 4). The infection rate was the lowest in the case of larvae (6.9%), and the highest in the case of imago (24.1%, Table 4). The exception was the infection of larvae at the Lubieszyn study site (53.3%) and nymphs at the Świerznica locality (40.1%, Table 4).

**Identification of *Borreliaceae* species in ticks collected in northern Poland.** Digestion of PCR product with enzyme HpyF3I and, if needed, PsuI, SatI or VspI allows for the identification of 14 *Borreliaceae* species (Table 5). The two most identified species were *Bl. afzelii* (29.4% of infected *I. ricinus*, including co-infections) and *Bl. garinii* (20.0% with co-infections, Table 5). The next was *Bl. spielmanii* (11.3% with co-infections) followed by *Bl. valaisiana* (9.4% including co-infections), *Bl. lanei* (7.7% with co-infections), *Bl. californiensis* (7.5% including co-infections), *B. miyamotoi* (6.3% including one co-infection), *Bl. burgdorferi* (6.0% with co-infections), *Bl. carolinensis* (3.1% including one co-infection), *Bl. americana* (1.9%), *B. turcica* (1.7% with co-infections), *Bl. lusitaniae* (1.4% including one co-infection), *Bl. bissettiae* (1.0%) and *Bl. finlandensis* (1.0% including one co-infection, Table 5). Out of the 415 infected ticks, 31 (7.5%) yielded mixed infections and as much as 23 comprised *Bl. garinii* and *Bl. afzelii* or included one of the mentioned species (Table 5).

Among 14 identified *Borreliaceae* species three were detected at all study sites, i.e., *Bl. afzelii*, *Bl. garinii* and *Bl. valaisiana*. Next, *Bl. spielmanii* was detected at six study sites, *Bl. burgdorferi*, *Bl. californiensis*, *Bl. lanei* and *Bl. miyamotoi* were detected at five study sites, whereas *Bl. americana* and *B. turcica* were detected at four, *Bl. lusitaniae* and *Bl. carolinensis* were detected at three, *Bl. finlandensis* was detected at two and *Bl. bissettiae* at individual study sites (Table 5). Out of the 14 identified species, five, *Bl. spielmanii*, *Bl. lanei*, *Bl. californiensis*, *Bl. finlandensis* and *B. turcica* were detected for the first time in the natural population of *I. ricinus* in Poland. Furthermore, *Bl. californiensis*, *Bl. lanei* and *B. turcica* were detected in field-collected *I. ricinus* for the first time in Europe.

Eight identified spirochete species, i.e., *Bl. garinii*, *Bl. afzelii*, *Bl. valaisiana*, *Bl. spielmanii*, *Bl. californiensis*, *Bl. lanei*, *Bl. americana*, and *B. miyamotoi* were detected in each developmental stage including larvae (Table 5). Four species were detected in two stages, i.e., *B. turcica* in nymphs and individual larva whereas *Bl. burgdorferi*, *Bl. lusitaniae* and *Bl. finlandensis* in imago and nymphs. *Bl. bissettiae* and *Bl. carolinensis* were detected only in nymphs (Table 5).

**Genetic diversity of *Borreliaceae* species detected in *I. ricinus*.** Analysis of 101 *flaB* gene fragment sequences and comparison with reference strains confirmed the genetic identity of each species identified using the PCR-RFLP protocol. The mean genetic distance within identified spirochete species ranged from 0.0 for *Bl. americana* and *Bl. lanei* to 0.0119 for *B. miyamotoi* (Table S1) whereas the distance between spirochete species ranged from 0.008 for *Bl. carolinensis* and *Bl. bissettiae* to 0.0711 for *Bl. finlandensis* and *Bl. spielmanii* inside the *Borreliella* genus and from 0.1588 for *Bl. valaisiana* and *B. turcica* to 0.1988 for *Bl. californiensis* and *B. miyamotoi* when individual *Borreliella* and *Borrelia* species were compared (Table S2). The distance between two identified *Borrelia* species came to 0.146 (Table S2). The highest diversity in the studied species was in *B. miyamotoi* when compared to strains from different continents (0.0119).

The genetic identity of the detected *Borreliaceae* species was also confirmed by analysis of 80 sequences of intergenic spacer between *mag* and *trnI* genes. The diversity ranges in this analysis including distances inside and between individual species was not comparable with those obtained on the basis of the *flaB* gene. The mean genetic distance within identified spirochete species ranged from 0.0 for *Bl. lusitaniae* to 0.0337 for *Bl. garinii* (Table S3) whereas the distance between spirochete species ranged from 0.032 for *Bl. burgdoeferi* and *Bl. finlandensis* to 0.2433 for *Bl. afzelii* and *Bl. bissettiae* inside the *Borreliella* genus and from 0.3754 for *Bl. americana* and *B. turcica* to 0.454 *Bl. afzelii* and *B. miyamotoi* when individual *Borreliella* and *Borrelia* species were compared (Table S4). The distance between two identified *Borrelia* species came to 0.2674 (Table S4). The highest diversity ever in the present study was in the case of *Bl. garinii* when compared to strains from Europe and Asia (0.0377).

**Figure 2:**
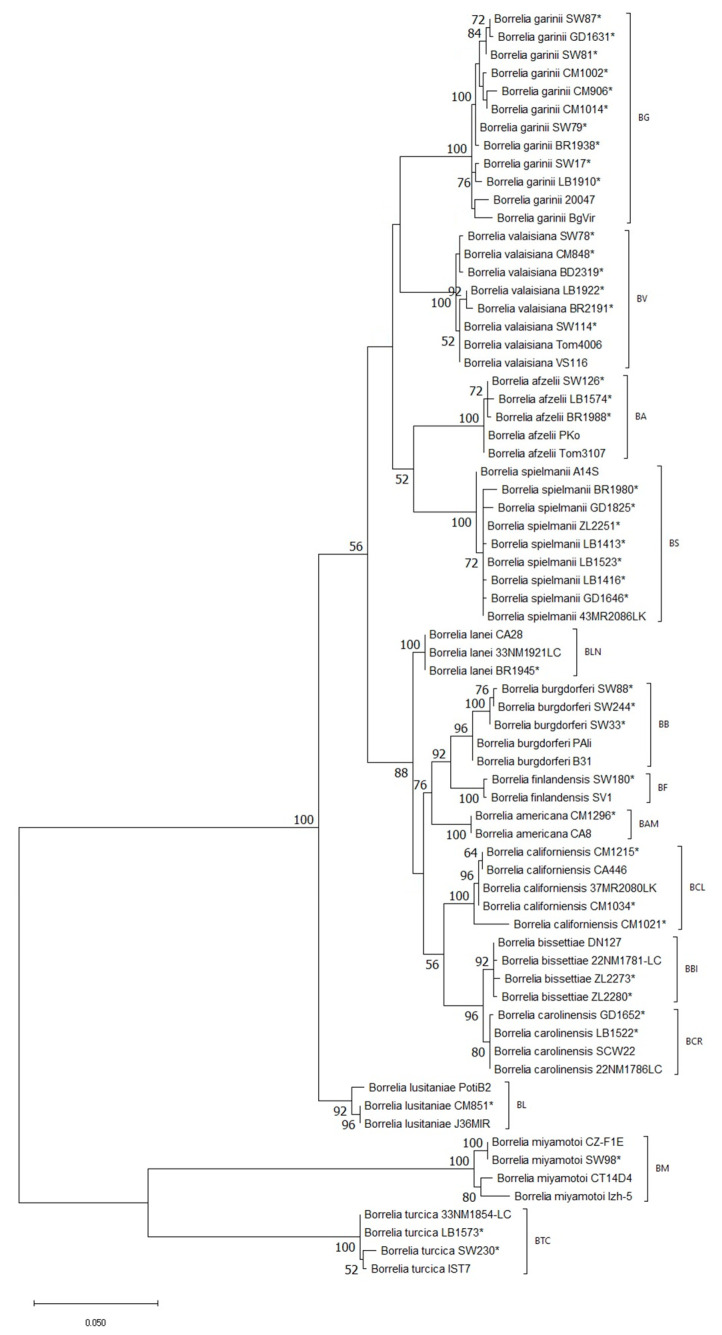
Phylogenetic analysis of *Borreliaceae* species obtained on the basis of *flaB* gene fragment (length 783–789 bp) amplified with primers FL120F and FL908R. The analysis involved 70 sequences. Bootstrap values >50 are shown. (*)—sequences obtained in this study. BG—*Borreliella garinii*, BA—*Bl. afzelii*, BB—*Bl. burgdorferi*, BV—*Bl. valaisiana*, BL—*Bl. lusitaniae*, BS—*Bl. spielmanii*, BBI—*Bl. bissettiae*, BF—*Bl. finlandensis*, BCL—*Bl. californiensis*, BCR—*Bl. carolinensis*, BLN—*Bl. lanei*, BAM—*Bl. americana*, BTC—*Borrelia turcica*, BM—*B. miyamotoi*.

**Figure 3:**
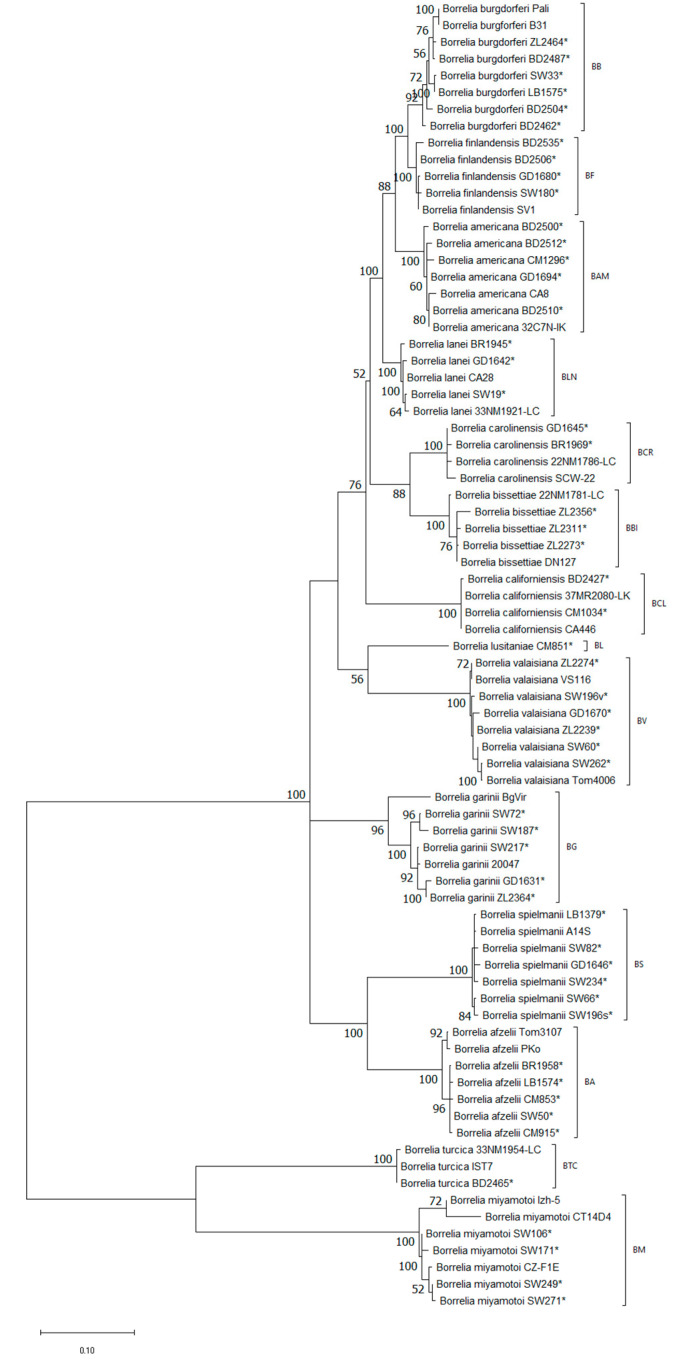
Phylogenetic analysis of *Borreliaceae* species obtained on the basis of *mag-trnI* intergenic spacer fragment (length 302–1176 bp) amplified with primers glz435f and ile20r. The analysis involved 78 sequences. Bootstrap values >50 are shown. (*)—sequences obtained in this study. BG—*Borreliella garinii*, BA—*Bl. afzelii*, BB—*Bl. burgdorferi*, BV—*Bl. valaisiana*, BL—*Bl. lusitaniae*, BS—*Bl. spielmanii*, BBI—*Bl. bissettiae*, BF—*Bl. finlandensis*, BCL—*Bl. californiensis*, BCR—*Bl. carolinensis*, BLN—*Bl. lanei*, BAM—*Bl. americana*, BTC—*Borrelia turcica*, BM—*B. miyamotoi*.

Phylogenetic analysis carried out on the basis of *flaB* gene sequences as well as *mag-trnI* intergenic spacer sequences confirmed the identity of each studied species as they grouped separately from each other giving independent branches on both dendrograms (Figure 2 and Figure 3). All twelve *Borreliella* species formed separate groups from species of the *Borrelia* genus, but relations between *Borreliella* species differed when the *flaB* gene and *mag-trnI* intergenic spacer were analyzed. The analysis of *flaB* gene sequences revealed three subgroups formed inside *Borreliella* according to their geographic range. Thus, one subgroup comprised three Eurasian species (*Bl. garinii*, *Bl. afzelii*, *Bl. valaisiana*) and one European (*Bl. spielmanii*), the second—six European and North American species (*Bl. burgdorferi*, *Bl. californiensis*, *Bl. carolinensis*, *Bl. bissettiae*, *Bl. lanei*, and *Bl. americana*) and one European (*Bl. finlandensis*), and the third *Bl. lusitaniae* found in Europe and North Africa (Figure 2). In the case of *Bl. californiensis* detected in this study for the first time in Europe, the obtained sequences represented different genotypes from the reference strain (Figure 2).

Contrary to the *flaB* gene analysis dendrogram established based on the *mag-trnI* intergenic spacer revealed different relations between Eurasian *Borreliella* species. They formed three groups, one comprised of *Bl. valaisiana* and *Bl. lusitaniae*, the second was formed separately by *Bl. garinii* and the third by *Bl. afzelii* and *Bl. spielmanii* (Figure 3).

## 4. Discussion

In Europe, the most frequent tick species and also the main vector of *Borreliaceae* spirochetes is the common tick *I. ricinus*. This tick species occurs in deciduous and mixed forests and an increasing number of human residential areas in the vicinity of the natural tick habitat and the continuing warming of the climate promote exposure to tick bites. The distribution range of *I. ricinus* reaches the north of Africa where a new morphological form of this species was detected with accompanying molecular diversity and classified as new species *I. inopinatus* [28]. The new species was also found in different European countries, i.e., Germany, Austria, Romania and Switzerland and represented 0.9–7.9% of the tick population [33,34,35,36,37,38,39,40]. Therefore molecular identification of ixodid ticks was the part of present work. The PCR-RFLP and sequencing analysis of mitochondrial *coxI* gene revealed, however, that except for one specimen of *Haemaphysalis concinna* nymph, the rest of the examined ticks were identified as *I. ricinus*. *H. concinna* and were found in western and southern Poland with limited incidence [41,42,43]. The first description of *H. concinna* was made in north-western Poland [44] near Zielonczyn, where the tick was found in the present study.

The DNA of *Borreliaceae* spirochetes was detected in 19.0% of *I. ricinus* and ranged at all collection sites between 8.3% and 32.6% (Table 3). The infection rate obtained in this study fits into the range between 3.7% and 38.1% established for the European tick populations examined in the last ten years including Poland [16,40,45,46,47,48,49,50,51,52,53].

So far, 16 *Borreliaceae* species have been detected in ixodid ticks in Europe including 12 species found in field-collected *I. ricinus*, i.e., *Borreliella afzelii*, *Bl. garinii*, *Bl. burgdorferi*, *Bl. valaisiana*, *Bl. lusitaniae*, *Bl. spielmanii*, *Bl. bissettiae*, *Bl. bavariensis*, *Bl. finlandensis*, *Bl. carolinensis*, *Bl. americana* and *Borrelia miyamotoi* [10,16,40,45,46,47,48,49,50,53]. Among the above mentioned only *Bl. finlandensis*, *Bl. carolinensis* and *Bl. americana* are not recognized as pathogenic [13,54,55]. Two other species, *Bl. turdi* and *Bl. californiensis* were detected in *I. ricinus* collected from birds and foxes, respectively [11,15]. The last two, *Bl. lanei*, and *B. turcica* were detected in ixodid tick species that specifically feed on bats and/or foxes [14,15]. A North American study of novel serological tests for the detection of *Borreliaceae* spirochetes points to the possible pathogenicity of the *B. californiensis* and *B. turcica* [25].

This precise identification of *Borreliaceae* spirochetes revealed 14 of 16 mentioned species with differential distribution and prevalence depending on distinct parts of Northern Poland. The most prevalent species in this study were *Bl. afzelii* and *Bl. garinii* (29.4% and 20% of infected ticks, respectively) and are the acclaimed predominant European *Borreliella* species including in Poland [16,40,47,49,50,51]. The next was *Bl. Spielmanii* (11.3%) an infrequent species in Europe and *Bl. Valaisiana* (9.4%), frequently detected in Germany, England, Slovakia, Moldova and Norway [16,40,45,47,50]. Unexpectedly, the next species were *Bl. lanei* and *Bl. californiensis* (7.7% and 7.5%, respectively). To the best of our knowledge, this is the first detection of these species in field-collected ticks from Poland and Europe. Two other species in the present study, i.e., *B. miyamotoi* (6.3%) and *Bl. burgdorferi* (6%) occur in many European countries including Poland [16,46,47,49,50,51,53]. On the contrary, the next two species, *Bl. carolinensis* (3.1%) and *Bl. americana* (2%) are detected in Europe only incidentally [7,10,56]. One of the rarest species in this study was *B. turcica* (1.7% of infected ticks). This species was originally isolated from the turtle-associated tick *Hyalomma aegyptium* [57] but recently it was found in *I. kaiseri* larva obtained from a red fox [15]. This is the third spirochete species in the present study detected for the first time in field-collected *I. ricinus*. The next species, *Bl. lusitaniae* (1.4%) is the main *Borreliella* species in southern Europe [16,48] and is relatively rare in the central and northern parts of the continent [16,40,46,47,49,50,51]. The last in this study, *Bl. bissettiae* and *Bl. finlandensis* (1% both) are detected incidentally in *I. ricinus* [16,46]. Specified incidence of *Borreliaceae* species include also co-infections (7.5% of infected ticks) that may become potential diagnostic difficulties, especially since three-quarters of detected mixed infections comprised *Bl. garinii* and/or *Bl. afzelii* acclaimed as the main pathogenic species in Europe [13,54]. Present results of mixed infections do not differ from other European studies that range from 1.2% to 13.6% [46,47,49,51].

Among the 14 identified spirochete species four of the most prevalent were found in every (*Bl. afzelii*, *Bl. garinii* and *Bl. valaisiana*) or almost every (*Bl. spielmanii*) study location. The remaining ten species were present on five (*Bl. bugdorferi*, *Bl. californiensis*, *Bl. lanei* and *B. miyamotoi*), four (*Bl. americana* and *B. turcica*), three (*Bl. lusitaniae* and *Bl. carolinensis*), two (*Bl. finlandensis*) or one study site (*Bl. bissettiae*) and statistically significant differences concerned the distribution of almost all species on particular study sites. The statistically significant differences were not observed only in the case of *Bl. lusitaniae*; the infection rate was similarly low on three study sites where it was detected. The presented study revealed a zonal distribution of different spirochete species from the *Borreliaceae* family in Northern Poland not only by the presence or absence of particular species but also by differences in infection rates that correlate with different risks of human and animal infection. Similar differences in the distribution and infection rates are observed in other European studies [45,46,47,51,58,59,60] but also in the case of different *Borreliaceae* species characteristics for Asia [61,62] and North America [63,64,65]. The cause of such zonality may be host specific, especially in the case of *Borreliella* species [66], and due to the differential availability in the studied area but not the climatic conditions which are similar for the entire area of research.

The analysis of variability of identified spirochete species revealed the differences in the case of both examined markers or only one of them (*Bl. lusitaniae*, *Bl. americana*, *Bl. lanei*). Each species demonstrated genetic distinctiveness from the others and genetic distance inside species was lower than the distance between the particular species. The distances inside and between species were also discernible on dendrograms established on the basis of the *flaB* gene and *mag-trnI* intergenic spacer. The diversity of *Borreliaceae* species noted in this study is also observed by other researchers [40]. It is, however, essential that in the present study the diversity was similar to that observed for the known European *Borreliella* species and was established for spirochete species detected for the first time in the natural populations of *I. ricinus*. This finding suggests the existence of newly detected spirochetes in Europe as the remaining European *Borreliella* species. *Borreliella californiensis* and *Bl. lanei* were detected for the first time in natural populations of *I. ricinus* and are closely related to *Bl. burgdorferi,* together with *Bl. bissettiae*, *Bl. carolinensis* and *Bl. americana* which are distributed in Europe and North America and also with *Bl. finlandensis* which is only from Europe and *Bl. kurtenbachii* which is detected only in North America [6]. According to the concept of *Borreliaceae* spirochetes evolution proposed by Estrada-Pena et al. [67], the common ancestor of the mentioned species might have existed in Laurasia before its split into North America and Eurasia.

It is worth to pay the attention to molecular markers used in this study for the detection of *Borreliaceae* DNA. In this study, markers were applied to allow for the detection of all the representatives of the *Borreliaceae* family in a nested PCR procedure with subsequent species identification by restriction analysis or by discrimination of the PCR product length. This procedure allows for avoiding results with undefined *Borreliella* species that occur in the case of using less variable molecular markers for standard PCR or species-specific probes for real-time PCR procedure [53,56,60,61,68]. The approach used in this study ensures identifying all PCR products so it allows for the assessment of the real representation of particular spirochete species in examined tick populations. Using such precise procedures is crucial for establishing the real distribution of *Borreliaceae* species, especially in light of the latest findings pointing to new potential spirochete species with possible medical importance [25].

## 5. Conclusions

The study of a natural population of *I. ricinus* in northern Poland proved the existence of the majority of the described European *Borreliella* species with the exclusion of two species that were described incidentally, i.e., *B. bavariensis* [31,51] and *B. turdi* [11]. The present study proved not only the predominant prevalence of *B. afzelii* and *B. garinii* in ticks from Poland [30,49,51,52] but also the existence of two North American *Borreliella* species in host-seeking ticks in Europe, namely *B. californiensis* and *B. lanei*. The detection of many *Borreliella* species in natural tick populations may affect the assessment of the risk of their transmission to humans especially as the present study concerned locations willingly visited by humans.

## Figures and Tables

**Table 1:** Primers used for the amplification DNA of Ixodidae ticks and *Borreliaceae* spirochetes.

Specificity	Genetic Marker	Sequence of Primers (5′->3′)	Annealing Temp. (°C)	Length of Amplicons (bp)	Usage	Reference
Ixodidae *	*coI*	CO1-45F: ACTAACCATAAAGACACATTGG	44	706	PCR-RFLP, sequencing	This study
		CO1-1100R: GAATTGGCTAAAATAATTCC				
		Nested PCR				
		CO1-375F: GGCAGGAACTGGATGAAC	47			
		CO1-1086R: AATTCCTGTTAATCCYCC				
Borreliaceae **	*flaB*	132f: TGGTATGGGAGTTTCTGG	56	604	PCR-RFLP	[24]
		905r: TCTGTCATTGTAGCATCTTT				
		Nested PCR				
		220f: CAGACAACAGAGGGAAAT	54			
		823r: TCAAGTCTATTTTGGAAAGCACC				
		FL84F: AGAAGCTTTCTAGTGGGTACAGA	57			
		FL976R: GATTGGCCTGTGCAATCAT				
		Nested PCR		789	sequencing	This study
		FL120F: TGATGATGCTGCTGGGATGG	56			
		FL908R: TCATCTGTCATTGTAGCATCTT				
	*mag—trnI*	glz199f—GTAAGTTTGCCAGGACCATT	56			
		ile20r—TGAACATCCGACCTCAGG				
		Nested PCR		309-1183	sequencing	This study
		glz435f—TAAGCTTCCGTTTCAAC	58			
		ile65r—CAGACCTGCGCTCTAACC				

* primers specific to the whole *Ixodes* genus. ** primers specific to the whole *Borreliaceae* family including Lyme disease borreliae (*Borreliella* genus), relapsing fever (RF) borreliae and reptile-related (REP) borreliae (*Borrelia* genus).

**Table 2:** Restriction patterns received after digestion of PCR product with primer set 220F and 823R specific for *Borreliaceae* spirochetes.

*Borreliaceae* Species	Restriction Fragment Size			
	HpyF3I	SatI	PsuI	VspI
*Bl. burgdorferi*	359, 207, 38	241, 200, 112, 51		
*Bl. finlandensis*	359, 207, 38	251, 226, 127		
*Bl. americana*	359, 207, 38	353, 200, 51		
*Bl. afzelii*	305, 165, 92, 42 *			
*Bl. garinii*	388, 135, 72, 9		327, 277	
*Bl. bavariensis*	388, 135, 72, 9		277, 219, 108	
*Bl. valaisiana*	188, 135, 117, 92, 72 *			
*Bl. lusitaniae*	305, 207, 92 *			
*Bl. bissettiae*	280, 135, 117, 72 *			
*Bl. spielmanii*	397, 207	241, 209, 112, 42		
*Bl. lanei*	397, 207	353, 200, 51		
*Bl. carolinensis*	280, 135, 117, 45, 27 *			
*Bl. californiensis*	280, 207, 117			461, 143
*Bl. turdi*	280, 207, 117			352, 143, 109
*B. miyamotoi*	512, 86 *			
*B. turcica*	412, 135, 45, 12 *			

* unique restriction pattern, further differentiation is not required.

**Table 3:** Reference strains of *Borreliaceae* spirochetes used in this study.

*Borreliaceae* Species	Strain	Source	Country	Accession Number	
				*flaB*	*mag-trnI*
*B. garinii*	20047	*I. ricinus*	France	CP018744	
	BgVir	*I. persulcatus*	Russia	CP003151	
*B. afzelii*	PKo	Skin biopsy	Germany	CP002933	
	Tom3107	*I. persulcatus*	Russia	CP009212	
*B. burgdorferi*	PAli	*Homo sapiens*	Germany	CP019844	
	B31	*I. scapularis*	USA	AE000783	
B. valaisiana	VS116	*I. ricinus*	Switzerland	ABCY02000001	
	Tom4006	*I. persulcatus*	Russia	CP009117	
*B. lusitaniae*	PotiB2	*I. ricinus*	Portugal	D82856	NA
	J3-6M-IR	*I. ricinus*	Poland	KF422805	NA
*Bl. bissettiae*	DN127	*I. pacificus*	USA	CP002746	
*B. spielmanii*	A14S	*Homo sapiens*	Netherlands	ABKB02000003	ABKB02000009
*B. finlandensis*	SV1	*I. ricinus*	Finland	ABJZ02000005	
*B. carolinensis*	SCW-22	*I. minor* fed on *Neotoma floridana*	USA	KF422810	MT119049
*B. californiensis*	CA446	*Dipodomys californicus*	USA	KF422809	MT119055
*B. lanei*	CA28	*I. pacificus*	USA	KF422812	MT119061
*B. americana*	CA8	*I. pacificus*	USA	KF422811	MT119066
*B. miyamotoi*	CT14D4	Human blood	USA	CP010308	
	Izh-5	Human blood	Russia	CP024205	
	CZ-F1E	*I. ricinus*	Czech Republic	CP046389	
*B. turcica*	IST7	*Hyalomma aegyptium* from tortoise	Turkey	CP028884	

NA—not accessible.

**Table 4:** Detection of *Borreliaceae* DNA in *I. ricinus* ticks collected from northern Poland.

Study Site	No. of Ticks Tested/Infected (%)				
	Total	Females	Males	Nymphs	Larvae
Świerznica	475/116 (24.4)	14/3 (21.4)	15/6 (40.0)	252/101 (40.1)	194/6 (3.1)
Ciemnik	509/42 (8.3)	20/4 (20.0)	29/1 (3.4)	276/24 (8.7)	184/13 (7.1)
Lubieszyn	306/40 (13.1)	16/1 (6.3)	26/3 (11.5)	249/28 (11.2)	15/8 (53.3)
Gdańsk	266/63 (23.7)	11/3 (27.3)	13/7 (53.8)	237/53 (22.4)	5/0 (0)
Bartoszewo	185/35 (18.9)	17/2 (11.8)	19/9 (47.4)	148/24 (16.2)	1/0 (0)
Zielonczyn	302/72 (23.8)	2/0 (0)	3/0 (0)	260/69 (26.5)	37/3 (8.1)
Bełdany Lake	144/47 (32.6)	16/8 (50)	27/8 (29.6)	101/31 (30.7)	
Total	2187/415 (19.0)	96/21 (21.9)	132/34 (25.8)	1523/330 (21.7)	436/30 (6.9)

**Table 5:** *Borreliaceae* species identified in *I. ricinus* from Northern Poland.

*Borreliaceae* Species	Study Site (PCR+ [F/M/N/L])							
	Lubieszyn	Bartoszewo	Zielonczyn	Ciemnik	Świerznica	Gdańsk	Bełdany Lake	Total (%)
*Bl. garinii*	9 [1/3/5/0]	8 [2/3/3/0]	4 [0/0/4/0]	7 [0/1/5/1]	35 [1/1/27/6]	5 [0/2/3/0]	3 [1/0/2/0]	71 [5/10/49/7] (17.1)
*Bl. afzelii*	5 [0/0/3/2]	7 [0/0/7/0]	30 [0/0/28/2]	10 [1/0/8/1]	38 [1/2/35/0]	3 [1/0/2/0]	10 [2/2/6/0]	103 [5/4/89/5] (24.8)
*Bl. burgdorferi*	1 [0/0/1/0]		3 [0/0/3/0]	1 [0/0/1/0]	7 [0/0/7/0]		5 [1/0/4/0]	17 [1/0/16/0] (4.1)
*Bl. valaisiana*	5 [0/0/4/1]	7 [0/2/5/0]	4 [0/0/4/0]	1 [1/0/0/0]	9 [1/1/7/0]	9 [0/0/9/0]		35 [2/3/29/1] (8.4)
*Bl. lusitaniae*			1 [0/0/1/0]	2 [2/0/0/0]	2 [0/0/2/0]			5 [2/0/3/0] (1.2)
*Bl. spielmanii*	12 [0/0/8/4]	4 [0/1/3/0]	1 [0/0/1/0]	3 [0/0/1/2]	3 [0/0/3/0]	22 [2/3/17/0]		45 [2/4/33/6] (10.8)
*Bl. bissettiae*			4 [0/0/4/0]					4 [0/0/4/0] (1.0)
*Bl. finlandensis*					1 [0/0/1/0]		2 [0/1/1/0]	3 [0/1/2/0] (0.7)
*Bl. californiensis*	1 [0/0/1/0]	1 [0/1/0/0]	8 [0/0/7/1]	4 [0/0/2/2]			13 [1/3/9/0]	27 [1/4/19/3] (6.5)
*Bl. carolinensis*	2 [0/0/2/0]	4 [0/0/4/0]				6 [0/0/6/0]		12 [0/0/12/0] (2.9)
*Bl. lanei*	3 [0/0/3/0]	1 [0/1/0/0]		5 [0/0/2/3]	2 [0/0/2/0]	14 [0/1/13/0]		25 [0/2/20/3] (6.0)
*Bl. americana*			2 [0/0/2/0]	1 [0/0/0/1]		1 [0/0/1/0]	4 [1/2/1/0]	8 [1/2/4/1] (1.9)
*B. miyamotoi*		2 [0/1/1/0]	8 [0/0/8/0]	3 [0/0/3/0]	9 [0/2/7/0]		3 [0/0/3/0]	25 [0/3/22/0] (6.0)
*B. turcica*	1 [0/0/0/1]				2 [0/0/2/0]		1 [0/0/1/0]	4 [0/0/3/1] (1.0)
*Bl. garinii/Bl. afzelii*			5 [0/0/5/0]		3 [0/0/3/0]			8 [0/0/8/0] (1.9)
*Bl. garinii/Bl. burgdorferi*					1 [0/0/1/0]			1 [0/0/1/0] (0.2)
*Bl. garinii/Bl. lanei*						1 [0/1/0/0]		1 [0/1/0/0] (0.2)
*Bl. garinii/Bl. lusitaniae*			1 [0/0/1/0]					1 [0/0/1/0] (0.2)
*Bl. garinii/Bl. valaisiana*			1 [0/0/1/0]					1 [0/0/1/0] (0.2)
*Bl. afzelii/Bl. burgdorferi*					2 [0/0/2/0]		1 [0/0/1/0]	3 [0/0/3/0] (0.7)
*Bl. afzelii/Bl. californiensis*		1 [0/0/1/0]					1 [1/0/0/0]	2 [1/0/1/0] (0.5)
*Bl. afzelii/Bl. carolinensis*						1 [0/0/1/0]		1 [0/0/1/0] (0.2)
*Bl. afzelii/Bl. lanei*				2 [0/0/0/2]		1 [0/0/1/0]		3 [0/0/1/2] (0.7)
*Bl. afzelii/B. turcica*	1 [0/0/1/0]							1 [0/0/1/0] (0.2)
*Bl. burgdorferi/Bl. valaisiana*					1 [0/0/1/0]		1 [1/0/0/0]	2 [1/0/1/0] (0.5)
*Bl. burgdorferi/Bl. californiensis*							1 [0/0/1/0]	1 [0/0/1/0] (0.2)
*Bl. burgdorferi/Bl. finlandensis*							1 [0/0/1/0]	1 [0/0/1/0] (0.2)
*Bl. valaisiana/Bl. spielmanii*					1 [0/0/1/0]			1 [0/0/1/0] (0.2)
*Bl. spielmanii/Bl. lanei*				1 [0/0/1/0]				1 [0/0/1/0] (0.2)
*Bl. californiensis/B. turcica*							1 [0/0/1/0]	1 [0/0/1/0] (0.2)
*Bl. lanei/B. miyamotoi*				1 [0/0/0/1]				1 [0/0/0/1] (0.2)
*Bl. afzelii/Bl. lanei/B. turcica*				1 [0/0/1/0]				1 [0/0/1/0] (0.2)
Total	40 [1/3/28/8]	35 [2/9/24/0]	72 [0/0/69/3]	42 [4/1/24/13]	116 [3/6/101/6]	63 [3/7/53/0]	47 [8/8/31/0]	415 [21/34/330/30] (100.0)

F—female, M—male, N—nymph, L—larva.

## Data Availability

All data generated or analyzed during this study are included in this published article and its supplementary information file. The accession numbers of DNA sequences obtained for ticks and bacteria are mentioned in Material and Methods and are available in the GenBank (https://ww.ncbi.nlm.nih.gov/nuccore, accessed on 28 November 2022).

## Supplementary Materials

The following supporting information can be downloaded at: https://www.mdpi.com/article/10.3390/life13040972/s1, Table S1: *flaB* gene mean genetic distance within individual *Borreliaceae* species detected in host seeking ticks from Northern Poland; Table S2: MEGA 11 results of mean distance between *Borreliaceae* species obtained on the basis of *flaB* gene sequence fragment comparison; Table S3: *mag-trnI* intergenic spaces mean genetic distance within individual *Borreliaceae* species detected in host seeking ticks from Northern Poland; Table S4: MEGA 11 results of mean distance between *Borreliaceae* species obtained on the basis of intergenic spacer (IGS) of 3-methyladenine glycosylase (*mag*) and tRNA-Ile (*trnI*) genes sequence fragment comparison.
