# Supplementary material for: Genetic Diversity of Borreliaceae Species Detected in Natural Populations of Ixodes ricinus Ticks in Northern Poland
Source: Life (Basel). 2023 Apr 9;13(4):972. doi: 10.3390/life13040972 (PMC10143352; doi:10.3390/life13040972)
Supplement: Supplementary file 1 [file life-13-00972-s001.zip › life-2288888-supplementary.pdf]

Table S1. *flaB* gene mean genetic distance within individual *Borreliaceae* species detected in host seeking ticks from Northern Poland.

Spirochete species	Mean genetic dinstance within species
Bl. garinii	0.0076
Bl.valaisiana	0.0035
Bl.afzelii	0.0023
Bl.spielmanii	0.0034
Bl.burgdorferi	0.0051
Bl.finlandensis	0.0025
Bl.americana	0.0
Bl.californiensis	0.0064
Bl.bissettiae	0.0025
Bl.carolinensis	0.0006
Bl.lanei	0.0
Bl.lusitaniae	0.0051
B.turcica	0.004
B.miyamotoi	0.0119

Table S2. MEGA 11 results of mean distance between *Borreliaceae* species obtained on the basis of *flaB* gene sequence fragment comparison.

	BG	BV	BA	BS	BB	BF	BAM	BCL	BBI	BCR	BLN	BL	BTC
Bl.valaisiana	0.0468												
Bl.afzelii	0.0539	0.0456											
Bl.spielmanii	0.0532	0.0538	0.0473										
Bl.burgdorferi	0.0653	0.0570	0.0627	0.0637									
Bl.finlandensis	0.0675	0.0606	0.0691	0.0711	0.0253								
Bl.americana	0.0567	0.0523	0.0621	0.0621	0.0290	0.0325							
Bl.californiensis	0.0619	0.0524	0.0606	0.0622	0.0334	0.0417	0.0355						
Bl.bissettiae	0.0549	0.0495	0.0536	0.0574	0.0389	0.0430	0.0423	0.0317					
Bl.carolinensis	0.0560	0.0472	0.0520	0.0522	0.0382	0.0447	0.0400	0.0303	0.0080				
Bl.lanei	0.0518	0.0445	0.0511	0.0525	0.0277	0.0285	0.0245	0.0289	0.0310	0.0287			
Bl.lusitaniae	0.0590	0.0571	0.0654	0.0587	0.0589	0.0612	0.0581	0.0562	0.0554	0.0555	0.0459		
B.turcica	0.1650	0.1588	0.1684	0.1778	0.1702	0.1709	0.1653	0.1664	0.1643	0.1640	0.1634	0.1599	
B.miyamotoi	0.1930	0.1813	0.1836	0.1908	0.1970	0.1984	0.1875	0.1988	0.1972	0.1955	0.1863	0.1730	0.1460

BG – *Borreliella garinii*, BV – *Bl. valaisiana*, BA – *Bl. afzelii*, BS – *Bl. spielmanii*, BB – *Bl. burgdorferi*, BF – *Bl. finlandensis*, BAM – *Bl. americana*, BCL – *Bl. californiensis*, BBI – *Bl. bissettiae*, BCR – *Bl. carolinensis*, BLN – *Bl. lanei*, BL – *Bl. lusitaniae*, BTC – *Borrelia turcica.*

Table S3. *mag-trnI* intergenic spaces mean genetic distance within individual *Borreliaceae* species detected in host seeking ticks from Northern Poland.

Spirochete species	Mean genetic dinstance within species
Bl. garinii	0.0377
Bl.valaisiana	0.0079
Bl.afzelii	0.011
Bl.spielmanii	0.009
Bl.burgdorferi	0.0123
Bl.finlandensis	0.0054
Bl.americana	0.0087
Bl.californiensis	0.0013
Bl.bissettiae	0.0112
Bl.carolinensis	0.009
Bl.lanei	0.008
Bl.lusitaniae	0.0
B.turcica	0.0022
B.miyamotoi	0.027

Table S4. MEGA 11 results of mean distance between *Borreliaceae* species obtained on the basis of intergenic spacer (IGS) of 3-methyladenine glycosylase (*mag*) and tRNA-Ile (*trnI*) genes sequence fragment comparison.

	BG	BV	BA	BS	BB	BF	BAM	BCL	BBI	BCR	BLN	BL	BTC
Bl.valaisiana	0.2002												
Bl.afzelii	0.2003	0.2185											
Bl.spielmanii	0.1938	0.2080	0.1555										
Bl.burgdorferi	0.1554	0.1662	0.2168	0.2168									
Bl.finlandensis	0.1496	0.1571	0.2040	0.2055	0.0320								
Bl.americana	0.1376	0.1724	0.2320	0.2245	0.0650	0.0569							
Bl.californiensis	0.1320	0.1524	0.1664	0.1663	0.1256	0.1102	0.1282						
Bl.bissettiae	0.1812	0.2103	0.2433	0.2218	0.1267	0.1209	0.1379	0.1226					
Bl.carolinensis	0.1557	0.1971	0.2287	0.1907	0.1014	0.0937	0.1172	0.1088	0.0836				
Bl.lanei	0.1448	0.1382	0.2096	0.1873	0.0635	0.0582	0.0583	0.0911	0.1175	0.1038			
Bl.lusitaniae	0.2164	0.1374	0.2216	0.2033	0.1218	0.1270	0.1318	0.1461	0.1667	0.1471	0.1244		
B.turcica	0.3784	0.4089	0.4075	0.4429	0.3806	0.3954	0.3754	0.4062	0.3768	0.4026	0.3804	0.4181	
B.miyamotoi	0.4259	0.4176	0.4540	0.4287	0.4098	0.3956	0.4007	0.4390	0.4357	0.4304	0.3940	0.4014	0.2674

BG – *Borreliella garinii*, BV – *Bl. valaisiana*, BA – *Bl. afzelii*, BS – *Bl. spielmanii*, BB – *Bl. burgdorferi*, BF – *Bl. finlandensis*, BAM – *Bl. americana*, BCL – *Bl. californiensis*, BBI – *Bl. bissettiae*, BCR – *Bl. carolinensis*, BLN – *Bl. lanei*, BL – *Bl. lusitaniae*, BTC – *Borrelia turcica.*
